# Reconstruction of Alzheimer's Disease Cell Model *In Vitro* via Extracted Peripheral Blood Molecular Cells from a Sporadic Patient

**DOI:** 10.1155/2020/8897494

**Published:** 2020-12-18

**Authors:** Sijun Liu, Yuying Zhao, Xiaoying Su, Chengcheng Zhou, Peifen Yang, Qiusan Lin, Shijun Li, Hanxu Tan, Qi Wang, Changjun Wang, Qingguang Wu

**Affiliations:** ^1^School of Pharmaceutical Sciences, Guangzhou University of Chinese Medicine, Guangzhou 510006, China; ^2^Beth Israel Deaconess Medical Center, Department of Surgery, Harvard Medical School, 02120, USA; ^3^Institute of Clinical Pharmacology, Guangzhou University of Chinese Medicine, Guangzhou 510006, China; ^4^Radiology of Chinese PLA General Hospital, Beijing 100853, China; ^5^Martinos Center of Harvard Medical School, MA 02129, USA; ^6^Dongzhimen Hospital, Beijing University of Chinese Medicine, 100029, China; ^7^Guangdong Geriatric Institute, Guangdong Provincial People's Hospital, Guangzhou 510080, China

## Abstract

The establishment of human-induced pluripotent stem cell (iPSC) models from sporadic Alzheimer's disease (sAD) patients is necessary and could potentially benefit research into disease etiology and therapeutic strategies. However, the development of sAD iPSC models is still limited due to the multifactorial nature of the disease. Here, we extracted peripheral blood mononuclear cells (PBMCs) from a patient with sAD and induced them into iPSC by introducing the Sendai virus expressing Oct3/4, Sox2, c-Myc, and Klf4, which were subsequently induced into neural cells to build the cell model of AD. Using alkaline phosphatase staining, immunofluorescence staining, karyotype analysis, reverse transcription-polymerase chain reaction (RT-PCR), and teratoma formation *in vitro*, we demonstrated that the iPSC derived from PMBCs (PBMC-iPSC) had a normal karyotype and potential to differentiate into three embryonic layers. Immunofluorescence staining and quantitative real-time polymerase chain reaction (qPCR) suggested that PBMC-iPSCs were successfully differentiated into neural cells. Detection of beta-amyloid protein oligomer (A*β*O), beta-amyloid protein 1-40 (A*β* 1-40), and beta-amyloid protein 1-42 (A*β* 1-42) indicated that the AD cell model was satisfactorily constructed *in vitro*. In conclusion, this study has successfully generated an AD cell model with pathological features of beta-amyloid peptide deposition using PBMC from a patient with sAD.

## 1. Introduction

Since first reported by Dr. Alzheimer in 1906, the recognition of Alzheimer's disease had been developed for nearly 110 years. Alzheimer's disease (AD) is a neurodegenerative disease with an insidious onset and progressive development. The clinical manifestations include impairment in cognitive functions, mental and behavioral disorders, and gradual degeneration in daily living abilities. The pathogenesis of AD is complex and has not been fully elucidated. A widely accepted theory by scientific researchers is that deposition of amyloid beta (A*β*) in brain tissue could be the core process of both the development and progression of AD [1–9].

The lack of an ideal disease model for AD has seriously hindered studies on its pathogenesis and treatment. Traditionally, AD models have not been based on human neurons or glial cells, and animal models of AD have yet to be validated [10–12]. Since the progress of human AD results from an interaction between genes, there is a high chance that the pathological type of human AD needs to be expressed from one or more corresponding genes in mice. However, it is difficult to utilize results from animal models to explain the underlying biochemical reactions and pathological mutations associated with human AD [13].

The emergence of induced pluripotent stem cells (iPSCs) solves this problem [14–17]. iPSC was first discovered by Yamanaka et al. in 2006. They introduced four retroviruses, Oct3/4, Sox2, c-Myc, and Klf4, into mouse fibroblasts and induced their transformation into iPSC [18]. It has been shown that the generated iPSC had the ability to differentiate and self-renew and that they were similar to embryonic stem cells (ESCs) in morphology, gene, embryoid body and teratoma generation ability, differentiation ability, and other aspects [19–21]. This discovery demonstrated that iPSCs are capable of replacing ESC, which enables the avoidance of ethical problems existing in experimental embryonic stem cell research [22–24].

So far, most of the research showed has mainly focused on iPSC models from patients with familial AD (fAD). In fact, sporadic AD (sAD) is more prevalent and represents over 90% of the AD cases in the population [25]. The enlargement of more sAD models is therefore vital for studying this neurodegenerative disorder and could potentially benefit research into disease etiology and development of therapeutic strategies. However, iPSC-based models for sAD have shown a high degree of variability and inconsistencies in terms of AD hallmarks [26]; developing iPSC models of sAD remains challenging.

In this study, PBMCs were extracted from a patient with sporadic AD and induced into iPSC by introducing Oct 3/4, Sox2, c-Myc, and Klf4. The multipotency of PBMC-iPSC was identified through alkaline phosphatase staining, immunofluorescence staining, karyotype analysis, RT-PCR, and teratoma formation. Subsequently, iPSCs were induced to differentiate into neurons, and immunofluorescence staining, qPCR, and the detection of beta-amyloid protein content were used to determine whether the AD cell model was successful [27–36].

## 2. Methods

### 2.1. Establishment of iPSC

An iPSC line (iPS-22-1 and iPS-15-5) was maintained and differentiated according to the following method. 4 mL of peripheral blood was extracted from the patient's blood sample to obtain PMBC (iPS-22-1 was derived from one sporadic AD patient, age 53 yr, female; iPS-15-5 was derived from a control participant, age 55 yr, female). The human protocols were implemented strictly on the basis of the Ethical Guidelines of the Declaration of Helsinki. The study was approved by the ethics committee of Guangdong Provincial People's Hospital. PBMCs were cultured with IMDM, 10% (*v*/*v*) fetal bovine serum (FBS), 1 mM glutamine, and 10 ng/mL G-CSF for 5 days. The CytoTune™-iPS 2.0 Sendai Reprogramming Kit and Invitrogen Kit building system were used for induction. After 2-3 weeks, the appearance of ES cell-like colonies was observed under a light microscope, ES-like clones were isolated, and iPSC culture medium (Lonza) was used for amplification. iPSCs were identified through immunofluorescence and alkaline phosphatase staining, determination of karyotypes, and teratoma formation.

### 2.2. Immunofluorescence Staining

Primary antibodies consisted of Nanog (cat no. Ab80892, Abcam), Oct4 (cat no. sc-5279, Santa Cruz), Sox2 (cat no. AF2018, R&D systems), TRA-1-60 (cat no. MAB4360, Millipore), Donkey anti-Mouse IgG 488 conjugate (cat no. A-21202, Invitrogen), Donkey anti-Goat IgG 594 conjugate (cat no. A-11058, Invitrogen), Donkey anti-Rabbit IgG 594 conjugate (cat no. A-21207, Invitrogen), and Goat anti-mouse IgM 488 conjugate (cat no. A-21042, Invitrogen). Neurons were incubated on a PDL-coated glass cell slide (Thermo Fisher Scientific, MA, USA). Finally, neurons were labeled with DAPI (Sigma-Aldrich, MO, USA) at room temperature for 10 minutes. Images were obtained with a Zeiss Meta Confocal microscope.

### 2.3. Alkaline Phosphatase Staining

Alkaline phosphatase staining was performed with a Leukocyte Alkaline Phosphatase Kit (Sigma-Aldrich, MO, USA), according to the manufacturer's protocol. Cells were washed once with PBS. 20 mL citric acid, 1 mL of dd H_2_O, and 1.5 mL acetone were fixed for 30 seconds. Cells were then washed once with dd H_2_O. The dye solution consisted of 31.3 *μ*L Fastblue, 1.5 mL dd H_2_O, and 62.5 *μ*L AS-MX. Light dyeing was avoided for 30 minutes. Images were obtained with a camera.

### 2.4. Determination of Karyotypes

We added 50 *μ*L/mL colcemid to the cell culture and incubated it for up to 4 hours. Cells were digested with EDTA at 37°C for 5 min. Precipitation was collected after centrifugation (1500 rpm, 10 min); 10 mL preheated KCL solution was added to resuspend the cells. The sample was incubated at 37°C for 25 min. Then, we added 1 mL fixing solution (methanol : glacial acetic acid = 3 : 1, ready to use), centrifuged at 2500 rpm for 10 minutes. 10 mL fixing solution was added to resuspend the cells at room temperature for 3 min. The cells were centrifuged at 2500 rpm for 10 min to collect precipitation. 100 *μ*L-200 *μ*L precooled fixative was added to resuspend the cells. After fixing, samples were transferred onto precleaned slides and placed in the oven at 65°C overnight to dry. Finally, the slides were placed in a staining solution preheated at 37°C (9 mL phosphate buffer and 1 mL Giemsa original solution). They were stained for 15 min, then washed and dried. Cells were then analyzed and counted under the microscope. A total of 20 randomly selected metaphase spreads were counted and analyzed.

### 2.5. Teratoma Formation

iPSCs were suspended at 5 × 10^7^ cell/mL in Matrigel solutions. 100 *μ*L of the cell suspension was subcutaneously injected into the groin region of nude mice (Jackson Laboratory). All experiments were approved by the University Animal Ethics Committee. After 6 weeks of injection, tumors were surgically dissected from the mice. Samples were fixed in PBS containing 4% formaldehyde, embedded in paraffin, and stained with HE. Finally, we observed the samples under a microscope and photographed them.

### 2.6. Real-Time Quantitative PCR (RT-qPCR)

RNA was extracted using the TRIzol reagent (Invitrogen, CA, USA). Firstly, the cells were incubated overnight at -20°C with 1 mL TRI Reagent. RNA was extracted using a Direct-zol RNA MiniPrep kit from ZYMO RESEARCH. Secondly, we used an EasyScript cDNA Synthesis Kit for reverse transcription (components: nuclease-free H_2_O 14.5 *μ*L, RNA 2 *μ*g, Oligo (dT) 10 *μ*M, random primers 10 *μ*M, gene-specific primer 10 *μ*M, and dNTP mix 10 mM). The reaction solution was mixed and ice bathed for 1 min after centrifugation. Thirdly, the reaction liquid was added (components: 5×RT Buffer, RNaseOFF Ribonuclease Inhibitor 40 U/*μ*L, and EasyScript™ RTase 200 U/*μ*L), following reverse transcription for 50 minutes at 42°C and inactivation of reverse transcriptase for 5 minutes at 85°C. The obtained DNA was stored at -20°C. Finally, GAPDH was used as a loading control; Sox2, Lin28, Oct4, Nanog, and Rex1 were amplified by PCR to detect the RNA expression of the gene.

### 2.7. Neuron Differentiation

N2B27 medium was added at the beginning of differentiation (day 0); SB431542 and LDN193189 were added at the same time to induce differentiation into neurons. After 12 days of differentiation, SB431542 and LDN193189 were removed and cultured in N2B27 medium only. After 22 days of differentiation, the medium was replaced with ordinary neuron culture medium. At this point, neuron protruding and migration were apparent. After 28 days of differentiation, neurons were collected and observed in the open field. Immunofluorescence and qPCR were used to detect differentiated neurons. As for immunofluorescence staining, primary antibodies consisted of MAP-2 (cat no. M4403, Sigma), TUj1 (cat no. 845502, Biolegend), NeuN (cat no. ab177487, Abcam), SATB2 (cat no. ab34735, Abcam), and TBR1 (cat no. ab31940, Abcam). And the operation is similar to 2.2.

### 2.8. Quantitative PCR

Quantitative PCR was performed with SYBR Premix Ex Taq (Takara, CA, USA) enzyme. Each treatment group contained three independent replicates of the reaction, and the DNA obtained by reverse transcription was diluted tenfold as a template. The transcript content of each component was standardized by glyceraldehyde-3-phosphate dehydrogenase (GAPDH), and the relative expression was analyzed and calculated by comparative CT.

### 2.9. Detection of Human Beta-Amyloid Protein

#### 2.9.1. Beta-Amyloid Protein 1-40

The A*β* 1-40 Elisa kit (Sigma-Aldrich, MO, USA) was used in this experiment. First, reagents are moved to room temperature (18-25°C) for at least 30 min. A standard well and sample well are set up, respectively. 100 *μ*L of the sample was added to each empty space and incubated at 37°C for 2 hours. The liquid was discarded. Then, the biotin-labeled antibody working solution was prepared, and three compound wells were set for each sample. The biotin-labeled antibody solution was diluted with biotin-labeled antibody diluent at 100-fold dilution. 100 *μ*L of biotin-labeled antibody working solution was added to each well and incubated at 37°C for 1 hour. The washing working liquid was prepared, and the concentrated washing solution was diluted with deionized water at 1 : 25. The board was washed three times. Then, the horseradish peroxidase-labeled avidin working solution was prepared. The horseradish peroxidase-labeled avidin solution was diluted with horseradish peroxidase-labeled avidin diluent according to 1 : 100 times. 100 *μ*L was added to each well and incubated at 37°C for 1 hour. The liquid in the well was discarded, and the plate was washed five times. 90 *μ*L of substrate solution was added to each well in order, and 15-30 min was developed at 37°C. 50 *μ*L of termination solution was added to each well in order to terminate the reaction. After the termination of the reaction, the optical density (OD value) of each hole was measured sequentially at 450 nm wavelength.

#### 2.9.2. Beta-Amyloid Protein 1-42

The A*β* 1-42 Elisa kit (Sigma-Aldrich, MO, USA) was used in this experiment. 100 *μ*L standard and sample are added to each well. The liquid was shaken off, and 100 *μ*L biotin-antibody (1x) was added per cell, at 37°C to incubate for 1 hour. It was washed with lotion three times. Then, 100 *μ*L HRP-avidin (1x) was added to each well and incubated at 37°C for 1 hour. Cleaning was repeated five times. Then, 90 *μ*L TMB substrate is added to each well. It was incubated at 37°C for 30 minutes. Light was avoided. Finally, 50 *μ*L terminating solution was added to each well, and the optical density of each hole was determined within 5 minutes using the enzyme labeling instrument.

#### 2.9.3. Beta-Amyloid Protein Oligomer

The A*β*O Elisa kit (Sigma-Aldrich, MO, USA) was used in this experiment. 50 *μ*L of standard sample and 50 *μ*L of sample to be tested (diluted 5 times) were added. It was incubated at 37°C for 30 minutes. Washing was repeated 5 times. Then, 50 *μ*L of enzyme-labeled reagent was added to each well, except for the blank well. It was incubated at 37°C for 30 minutes. Washing was repeated 5 times. Then, the chromogenic agent A 50 *μ*L was added to each well, and then, the chromogenic agent B 50 *μ*L was added to avoid light at 37°C for 10 minutes. Finally, 50 *μ*L of terminating liquid was added to each well to terminate the reaction (blue turned to yellow). The absorbance (OD value) of each well was measured at 450 nm wavelength.

### 2.10. Statistical Analysis

One- and two-way ANOVAs were used to compare data between groups. Data are given as means ± SD. The significance threshold was *P* < 0.05. All statistical analyses were performed using SPSS version 25.0 (SPSS, Inc., USA).

## 3. Results

### 3.1. iPSC Derived from SAD

#### 3.1.1. Morphology of PBMC and iPSC

**Figure 1:**
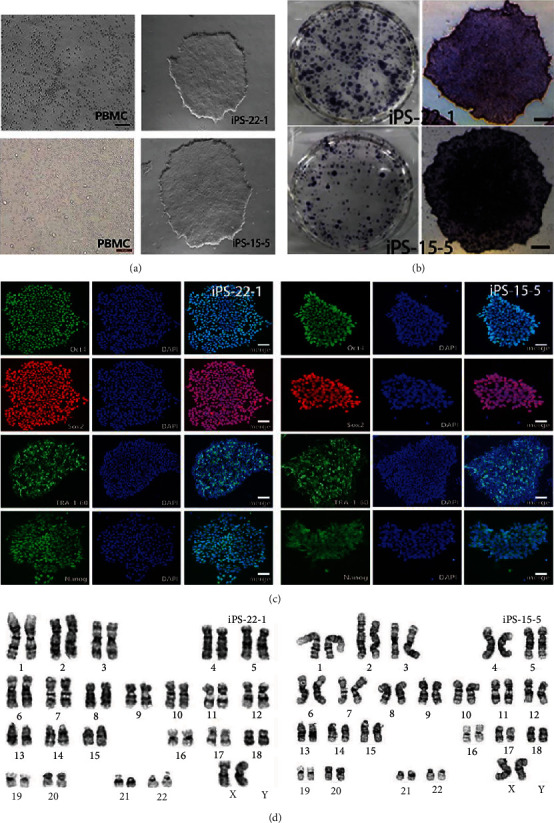
iPSC derived from SAD. iPSCs are similar to embryonic stem cells (ESCs) in morphology (a), which are characterized by colony-like growth, compact and tidy edges, small size, and high nuclear-cytoplasmic ratio. AP staining of iPSC-22-1 and iPSC-15-5 is positive (b). Specific protein expression of ESC was used to detect the immunofluorescence reaction of iPSC (c). The karyotype of iPSC was detected by karyotype analysis (d). The karyotypes of iPS-22-1 were 46, XX and those of iPS-15-5 were 46, XX. Both strains were normal cell karyotypes.

iPSCs are similar to embryonic stem cells (ESCs) in morphology (Figure 1(a)), which are characterized by colony-like growth, compact and tidy edges, small size, and a high nuclear-cytoplasmic ratio [16].

#### 3.1.2. Alkaline Phosphatase Staining and Immunofluorescence Staining

Alkaline phosphatase staining is a reliable method to identify the differentiated state of iPSC. The undifferentiated iPSC can be stained with dark purple. AP staining of iPSC-22-1 and iPSC-15-5 was positive (Figure 1(b)), indicating that the two strains of cells were in an undifferentiated state.

Specific protein expression of ESC was used to detect the immunofluorescence reaction of iPSC (Figure 1(c)). Cell staining was positive, indicating that iPSC expressed ESC specific protein. According to the results of immunofluorescence, iPSC from peripheral blood expressed the specific proteins of these stem cells.

#### 3.1.3. Karyotype Analysis

Due to the risk of karyotype abnormality in the process of iPSC line establishment, the probability of obtaining normal karyotype cells by using exogenous gene integration was rather low. Therefore, a Sendai virus vector (from Thermo Fisher's kit) was used to improve the probability of obtaining normal karyotype iPSC. The karyotype of iPSC was detected by karyotype analysis (Figure 1(d)). The results showed that the karyotypes of iPS-22-1 were 46, XX, and those of iPS-15-5 were 46, XX. Both strains were normal cell karyotypes.

#### 3.1.4. Real-Time Quantitative PCR (RT-qPCR)

**Figure 2:**
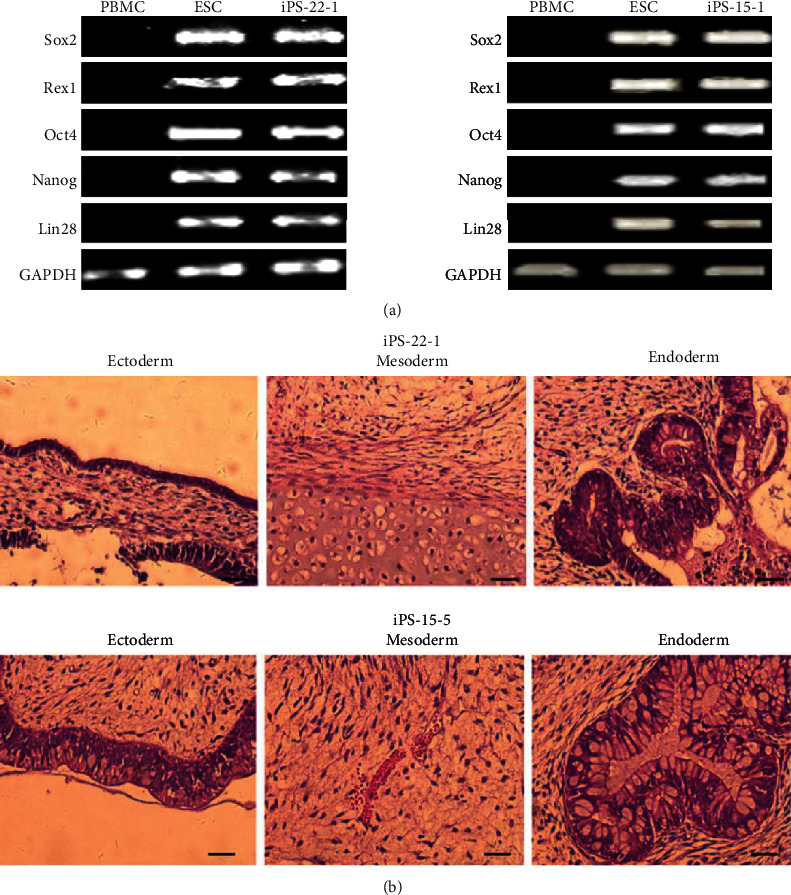
The results of RT-PCR and teratoma formation. The expressions of Oct4, Sox2, Nanog, Rexl, Lin28, and other genes were identified by RT-PCR. Sox2, Rex1, Oct4, Nanog, and Lin28 gene of two strains of cells were positive in agarose gel electrophoresis, which was similar to H9 cells, but negative in primary PBMC (a). The formation of teratoma was observed in vivo, and its ability of multidirectional differentiation was tested. iPSCs were tested for tumorigenesis in nude mice. The two iPSCs had the potential to differentiate into three layers (b).

The expression of exogenous genes in iPSC should be gradually reduced or silenced with the completion of reprogramming, and the endogenous gene expression should be activated. The selection of genes was based on the distinctive markers of ESC. The expressions of Oct4, Sox2, Nanog, Rexl, Lin28, and other genes were identified using RT-PCR (Figure 2(a)). The Sox2, Rex1, Oct4, Nanog, and Lin28 genes of two strains of cells were positive in agarose gel electrophoresis, which was similar to H9 cells, but negative in primary PBMC.

#### 3.1.5. Teratoma Formation

The formation of teratoma was observed *in vivo*, and its multidirectional differentiation ability was tested. iPSCs were tested for tumorigenesis in nude mice. 1 × 10^6^ ~ 5 × 10^6^ iPSCs were subcutaneously injected into nude mice, and the changes of teratoma were observed after a period of growth. The tumor tissue was removed and examined using HE staining. The results showed that the two iPSCs had the potential to differentiate into three layers (Figure 2(b)).

### 3.2. AD Modeling

#### 3.2.1. Differentiation of iPSC

**Figure 3:**
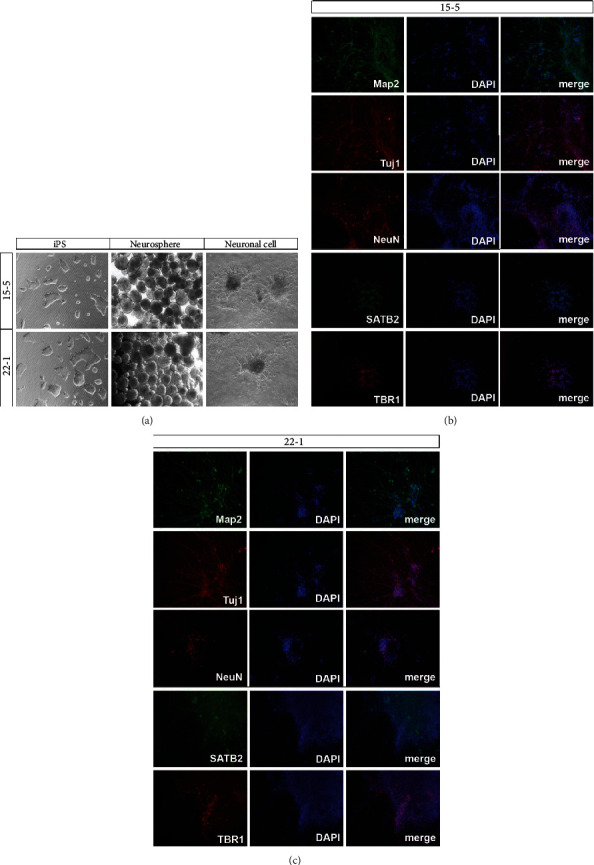
The result of differentiation. A large number of neurospheres could be observed under the microscope (a). After 28 days of differentiation and cultivation, the neuronal markers Tuj1, Map2, and NeuN and cortical neuronal markers SATB2 and TBR1 were detected by immunofluorescence, which showed that Tuj1, Map2, NeuN, SATB2, and TBR1 were positively expressed (b, c). *n* = 3.

Cortical neurons from iPS-22-1 and iPS-15-5 were differentiated from sporadic AD patients and from normal people (NC-PBMC-IPSC), respectively. We used the differentiation method described in Li et al. [20]. In general, iPSCs were cultured in N2B27 medium and induced to differentiate into neuron precursor cells by adding 2 *μ*M SB431542 and 100 nM LDN193189 SMAD signaling pathway inhibitors. After 12 days, cells were digested and cultured suspensive with N2B27 for 10 days. On the 22nd day of differentiation, a large number of neurospheres could be observed under the microscope (Figure 3(a)). In this case, the obtained neurospheres were cultured adherently with a neuron culture medium (component: neurobasal, 1x N2 supplement, 1x B27, GDNF 10 ng/mL, BDNF 10 ng/mL, CAMP 1 *μ*M, and L-ascorbic acid 0.2 mM). On the 23rd day, obvious neuron dendrites could be seen extending out of the neurosphere. Meanwhile, a large number of neurons were differentiated on the 28th day. Neuronal dendrites and axons extended out of the sphere. There was no significant difference between neurons derived from the sporadic AD patient and those derived from the control participant.

#### 3.2.2. Immunofluorescence

After 28 days of differentiation and cultivation, the neuronal markers Tuj1, Map2, and NeuN and cortical neuronal markers SATB2 and TBR1 were detected using immunofluorescence, which showed that Tuj1, Map2, NeuN, SATB2, and TBR1 were positively expressed after 28 days (Figures 3(b) and 3(c)). These results suggested that both types of iPSC had successfully differentiated into cortical neurons.

#### 3.2.3. qPCR

**Figure 4:**
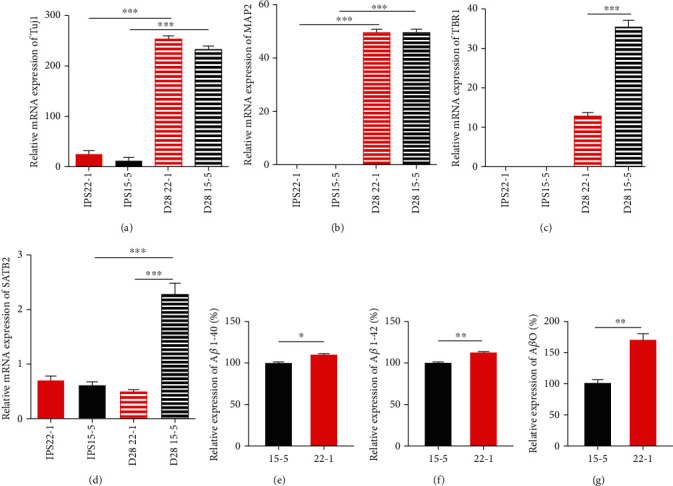
The qPCR results and the determination of beta-amyloid protein. After 28 days of culturing, Tuj1 and Map2 significantly increased. But there was no significant difference in expression of neuronal markers Tuj1 and Map2 between iPS-22-1 and iPS-15-5 cell (a, b). However, the expression of cortical neuron markers SATB2 and TBR1 of iPS-15-5 was higher than that of iPS-22-1 (c, d). ^∗^*P* < 0.05, ^∗∗^*P* > 0.05, and ^∗∗∗^*P* < 0.01. *n* = 3. The contents of A*β* 1-40 and A*β* 1-42 in adherent culture of neurons in AD patients had been higher than those in normal people (e, f). ^∗^*P* < 0.05, ^∗∗^*P* < 0.01. *n* = 3. The content of A*β*O in adherent culture solution of neurons in AD patients had been higher than that of normal people (g). ^∗^*P* < 0.05. *n* = 3.

On the 28th day of culturing, neuronal markers Tuj1 and Map2 and the cortical neuronal markers SATB2 and TBR1 were detected using qPCR (Figures 4(a) and 4(b)). The results showed that neuronal markers Tuj1 and Map2 had little or no expression in iPSC. After 28 days of culturing, Tuj1 and Map2 significantly increased. Comparing two strains of cell lines, we observed that there was no significant difference in the expression of neuronal markers Tuj1 and Map2 between iPS-22-1 and iPS-15-5 cells. However, the expression of cortical neuron markers SATB2 and TBR1 was higher for iPS-15-5 than iPS-22-1 (Figures 4(c) and 4(d)). The results showed that there was no significant difference between iPS-22-1 and iPS-15-5 in differentiating into neurons, but the efficiency of iPS-22-1 was lower in differentiating into cortical neurons than iPS-15-5.

#### 3.2.4. Determination of Beta-Amyloid Protein

A*β* 1-40 and A*β* 1-42 were detected in the neurons of the control participant and compared with A*β* 1-40 and A*β* 1-40 in the neurons of the patient with sporadic AD (Figures 4(e) and 4(f)). The levels of A*β* 1-40 and A*β* 1-42 in the adherent culture of neurons in the AD patient were higher than those in the control participant, with statistically significant differences.

The concentration of A*β* oligomer (A*β*O) in the sample was calculated and compared between the control and sporadic AD patients (Figure 4(g)). The levels of A*β*O in adherent culture solution of neurons were significantly higher in the AD patient than in the control participant.

## 4. Discussions

In AD, early pathological changes can be observed in the brain. Therefore, an ideal disease model of AD is needed for research. It is difficult to correlate genetic expressions observed in animal models to human AD, including biochemical reactions and pathological processes. Numerous drugs have shown significant efficacy in animal models, but not in clinical trials [37–41]. The lack of an ideal disease model of AD therefore has seriously hindered research on the pathogenesis and treatment of AD.

The emergence of iPSC provides a new way to solve this problem. We extracted peripheral blood cells from patients and induced them into iPSC and subsequently induced them into neurons. These neurons have the pathological manifestations of AD. The current study therefore provides a novel model of AD that can be used for the investigation of the pathological mechanism of AD and drug screening [41]. We regarded peripheral mononuclear blood cells as somatic cells. Compared with other somatic cells, peripheral mononuclear blood cells have numerous advantages, such as convenient sourcing, less trauma, shorter induction time *in vitro*, and the least possibility of mutation during induction. Moreover, the patients' peripheral blood-derived iPSC had specificity and no immunorejection [42, 43].

iPSCs are similar to ESC in morphology and have the ability of self-renewal and infinite differentiation. PBMC-derived iPSC expressed ESC specific marker molecules, and the endogenous pluripotent gene expression profile was similar to ESC, with the ability of multidirectional differentiation. The main findings are as follows: (1) For the detection of gene expression, the endogenous pluripotent genes Nanog, Oct4, Rexl, and Sox2 in iPSC and ESCs were similar but significantly higher than those in PBMCs before programming. (2) For karyotype analysis, both strains of iPSC had normal karyotypes, 46 chromosomes and XX type, indicating that PBMCs still maintained normal karyotypes after being induced into iPSCs *in vitro*. (3) AP staining and immunofluorescence staining were positive, indicating that the cells were undifferentiated and expressed ES cell-specific membrane proteins (SSEA3, SSEA4), plasma proteins (TRA 1 1-60, TRA 1 1-81), and nucleoprotein (Nanog). (4) For *in vivo* differentiation of teratoma, iPSCs were capable of forming teratoma entities in SCID mice, which contain tissue cells from the endoderm, mesoderm, and ectoderm.

After inducing iPSC into neurons, immunofluorescence and qPCR detection showed that both cells could effectively differentiate into cortical neurons and that they expressed neuronal markers Tuj1, Map2, SATB2, and TBR1. In the development mechanism of AD, proteolysis of amyloid precursor protein (APP), which first cleaves extracellularly by b-secretase and then within the membrane by g-secretase, produces beta-amyloid peptides (A*β*). A*β* peptides accumulate in the brain to form amyloid plaques; thus, it is a hallmark of AD [44]. However, a previous study showed that the levels of these markers for the other sAD patient-derived neurons were again close to those observed in nondemented controls [33]. No statistically significant change in the secretion of A*β*1-40, A*β*1-42, and the ratio A*β*1-42/A*β*1-40 was observed for sAD-derived neural models compared to controls [45, 46]. Instead, in our study, the expression of A*β*O, A*β*1-40, and A*β*1-42 in the sAD patient-derived neurons was significantly higher than in the control participant, as assessed by the detection of neuron A*β* oligomers, suggesting that the cell model of patients had typical pathological changes of sAD. In the future, our cell model could be used to assess interventions for sAD and further study the pathological mechanism of sAD.

## 5. Conclusions

Peripheral blood mononuclear cells are easier to obtain, with less trauma, shorter induction time *in vitro*, and minimal possibility of mutation in the induction process compared with other types of somatic cells. In addition, iPSC from patients' peripheral blood has specificity and causes no immunorejection. We found that iPSC and ESC have the same morphology. They have transcription factors Oct4, Sox2, Rex1, and Lin28 and nuclear protein (Nanog) and plasmin (TRA-60) which are indispensable when ESC expresses totipotency. At the same time, iPSCs were implanted subcutaneously in mice and resulted in multiple tissues containing three layers of germ cells in the tumors.

The AD disease model induced by sporadic AD-PMBC-iPSC successfully expressed beta amyloid peptide deposition. The model induced by cells obtained from a patient with sAD was more specific and representative. This model is promising for research on the pathogenesis of AD, drug screening, and drug toxicity evaluation.

## Data Availability

The experimental data used to support the findings of this study are included within the article.
